# Prospects of GWAS and predictive breeding for European winter wheat’s grain protein content, grain starch content, and grain hardness

**DOI:** 10.1038/s41598-020-69381-5

**Published:** 2020-07-27

**Authors:** Quddoos H. Muqaddasi, Jonathan Brassac, Erhard Ebmeyer, Sonja Kollers, Viktor Korzun, Odile Argillier, Gunther Stiewe, Jörg Plieske, Martin W. Ganal, Marion S. Röder

**Affiliations:** 1 Leibniz Institute of Plant Genetics and Crop Plant Research (IPK), Corrensstraße 3, 06466 Stadt Seeland OT Gatersleben, Germany; 2 KWS LOCHOW GmbH, 29303 Bergen, Germany; 3 Syngenta France S.A.S, 78910 Orgerus, France; 4 Syngenta Seeds GmbH, 32107 Bad Salzuflen, Germany; 5 TraitGenetics GmbH, Am Schwabeplan 1b, 06466 Stadt Seeland OT Gatersleben, Germany; 6 Present Address: European Wheat Breeding Center, BASF Agricultural Solutions GmbH, Am Schwabeplan 8, 06466 Stadt Seeland OT Gatersleben, Germany; 7 Present Address: SaKa Beteiligungsgesellschaft mbH, Albert-Einstein-Ring 5, 22761 Hamburg, Germany

**Keywords:** Plant genetics, Plant breeding, Agricultural genetics

## Abstract

Grain quality traits determine the classification of registered wheat (*Triticum aestivum* L.) varieties. Although environmental factors and crop management practices exert a considerable influence on wheat quality traits, a significant proportion of the variance is attributed to the genetic factors. To identify the underlying genetic factors of wheat quality parameters viz., grain protein content (GPC), grain starch content (GSC), and grain hardness (GH), we evaluated 372 diverse European wheat varieties in replicated field trials in up to eight environments. We observed that all of the investigated traits hold a wide and significant genetic variation, and a significant negative correlation exists between GPC and GSC *plus* grain yield. Our association analyses based on 26,694 high-quality single nucleotide polymorphic markers revealed a strong quantitative genetic nature of GPC and GSC with associations on groups 2, 3, and 6 chromosomes. The identification of known *Puroindoline-b* gene for GH provided a positive analytic proof for our studies. We report that a locus *QGpc.ipk-6A* controls both GPC and GSC with opposite allelic effects. Based on wheat's reference and pan-genome sequences, the physical characterization of two loci viz., *QGpc.ipk-2B* and *QGpc.ipk-6A* facilitated the identification of the candidate genes for GPC. Furthermore, by exploiting additive and epistatic interactions of loci, we evaluated the prospects of predictive breeding for the investigated traits that suggested its efficient use in the breeding programs.

## Introduction

Quality traits have a significant bearing on the end-use and monetary value of the wheat grain. In Germany, bread wheat varieties are registered at the Federal Plant Variety Office (Bundessortenamt) according to four classes. Based on the quality parameters, the wheat varieties are classified as (1) E-class, i.e., elite quality, (2) A-class, i.e., A-quality, (3) B-class, i.e., bread making, and (4) C-class, i.e., used for stock-feed purposes (https://www.bundessortenamt.de). Together with the sedimentation values (e.g., SDS and Zeleny tests), falling number and minerals, protein content, starch content, and grain hardness are among the leading parameters that form the basis of quality criteria of wheat.

The wheat grain protein content (GPC) influence gliadin to glutenin ratio that eventually govern the viscoelastic properties and bread-making quality of dough^1,2^. High protein wheat ensures maximum dividends for the farmers and low protein wheat—especially in the years of high protein discounts and premium—imposes substantial financial losses on the producers^3^. Coupled with grain yield (GY), improving the GPC is, therefore, one of the principal targets of virtually every wheat breeding program. Nevertheless, there exists a tight negative correlation between the wheat GPC and GY^4,5^. The GPC is influenced by the ambient environmental factors such as annual precipitation, crop rotation and cultivation practices, and soil fertility management systems, e.g., application of artificial nitrogen fertilizers^6–8^. However, GPC in wheat varieties is also typically regarded as a quantitative trait: it is controlled by a concerted action of several small- to medium-effect genetic loci. In addition to crop management practices, identification of trait-underlying loci is deemed a sustainable agricultural strategy to improve the genetic gains over time. For this purpose, several studies described quantitative trait loci (QTL) for GPC in bi-parental or diverse mapping populations in hexaploid^9–14^, tetraploid^15–18^, and hybrid wheat^19–21^. Little is, however, known about the genes determining the inheritance of GPC in wheat. A map-based cloning approach identified a NAC [for NAM (NO APICAL MERISTEM), ATAF1/ATAF2 (*Arabidopsis thaliana* ACTIVATING FACTOR), and CUC1/CUC2 (CUP-SHAPED COTYLEDON)]^22,23^ transcription factor (NAM-B1) as a causative gene underlying high GPC locus (*GPC-B1*) that originated from the wild emmer wheat *Triticum turgidum* ssp. *dicoccoides*^24,25^. The wild emmer wheat allele accelerates the senescence and results in (1) increased nutrient remobilization from leaves to the developing grains, and (2) increased grain protein, iron, and zinc content.

Starch is a major storage component of wheat grain endosperm. Being composed of two polymers viz., amylose, and amylopectin in the ratio of 1:3, the grain starch content (GSC) affects the end-use value of bread, e.g., dough rheology, bread staling, and crumb structure^26,27^. The GSC positively correlates with the increase in grain size and, eventually, GY^28^. Besides quantity, the quality of starch granules (physicochemical properties) helps determine the flour quality, flour yield, and water retention capacity of dough—a vital trait that influences the loaf volume. The GSC, however, shows negative correlation with the GPC. This warrants the fine-tuning of GPC and GSC in experimental lines to maintain the right protein levels while keeping high GY^29,30^. Although the genetic nature of GSC is less quantitative than GPC, similar to GPC, genes determining the inheritance of GSC in bread wheat varieties are unknown. A major QTL controlling the B-type starch granule content was discovered^31,32^ in wild *Aegilops* species. Recently, the causative gene was identified as an ortholog of the rice gene *FLOURY ENDOSPERM 6* coding for an unknown protein containing a CBM48 domain^33,34^.

Grain hardness (GH) of wheat—primarily determined by the endosperm texture—is a physical quality trait that is used for market grading. The world wheat trade is largely carried out based on the GH grades. Based on the endosperm texture, wheat is generally graded into soft, medium-soft, medium-hard, hard, and extra-hard types^35^. Softer wheat kernels are easily fractured and result in a large number of intact starch granules, whereas, harder types need relatively more power-consumption to mill and, therefore, produce coarser and damaged starch granules^36^. The wheat GH is distinguished by the expression of a major hardness (*Ha*) locus located on chromosome 5DS^37–39^. The *Ha* locus harbors the genes that encode 15-kD marker protein called friabilins that determine wheat softness. The friabilins are composed of a mixture of two lipid-binding puroindoline a and b (pinA and pinB) polypeptides^38^. It was demonstrated that mutations in the *Pin* genes control the hardness or softness in wheat grains^39^. Several alleles of the *Pinb-D1* locus are known and were characterized in wheat varieties^40^.

Since most of the wheat quality parameters harbor a quantitative genetic architecture, genome-wide prediction—based on high-density molecular markers—to predict the total genetic value of a trait becomes a method of choice in applied breeding programs^41^. Recently, genome-wide prediction on wheat populations of diverse genetic backgrounds suggested that wheat quality traits can be predicted with high accuracy^12,20,21,42,43^.

Here, we evaluated GPC, GSC, and GH in a panel of registered European winter wheat varieties in field trials. Our analyses showed that a high and significant genetic variation exists for wheat grain quality traits. Our association analyses with high-density single nucleotide polymorphism arrays revealed a quantitative genetic architecture with a few loci being significantly associated with the investigated traits. Exploiting wheat’s reference genome sequence^44^ and genomic diversity across varieties sequenced within the framework of wheat pan-genome, we identified putative candidate genes determining the inheritance of the investigated traits. We also studied the prospects of predictive breeding, and the results suggested that genomic selection can be performed to improve the genetic gains for wheat quality traits.

## Results

### Phenotypic data analyses reveal significant genetic variation, high heritability, and high correlation among wheat grain quality traits

**Figure 1:**
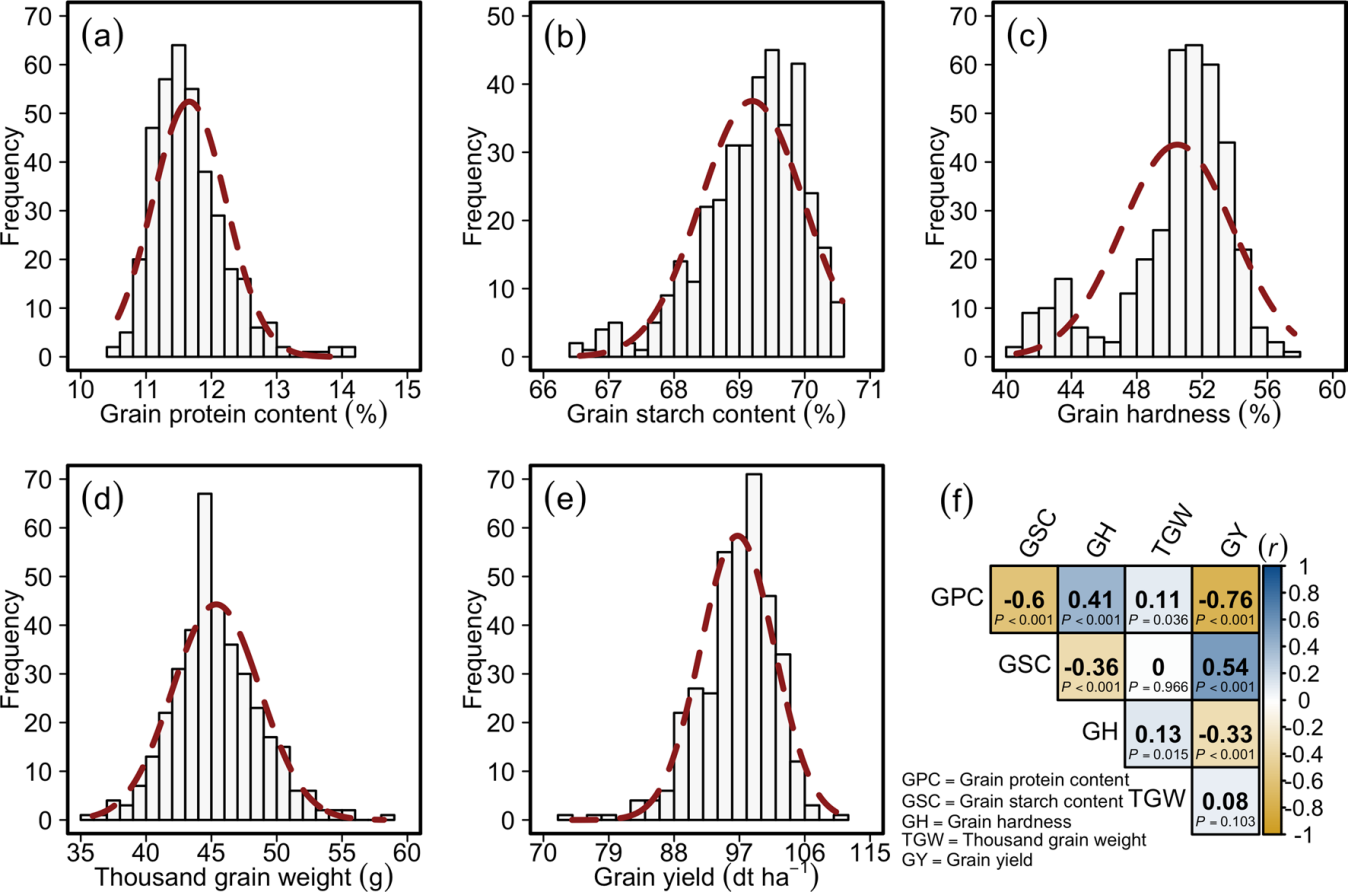
Phenotypic distribution and correlation of the investigated traits in a panel of 372 wheat varieties. Distribution of (**a**) grain protein content (%), (**b**) grain starch content (%), (**c**) grain hardness (%), (**d**) thousand-grain weight (g) and (**e**) grain yield (dt ha^-1^); (**f**) Pearson’s product-moment correlation $(r)$ among the investigated traits. *P*-value denotes the significance of the respective correlation.

The assessment of three wheat grain quality traits viz., grain protein content (GPC (%)), grain starch content (GSC (%)), and grain hardness (GH (%)) was performed in replicated trials in three to eight environments (Table 1) on a set of 372 (358 winter type; 14 spring type) wheat varieties registered for European markets (Table S1). We observed a significant genotypic variance, consistent performance, and positive average Pearson's product-moment correlation ($\stackrel{-}{r}$ = 0.57–0.75) across all the environments for the investigated traits (Fig. S1a–c; Tables S2a–c and Table S3a–c). Since quality traits are reported to influence thousand-grain weight (TGW (g)) and grain yield (GY ($dt {ha}^{-1}$)), we evaluated the grain quality data against multi-environment TGW and GY data taken from a previous study^45^. The distribution of the best linear unbiased estimations (BLUEs) calculated across environments showed a wide genotypic variation in all of the investigated traits (Fig. 1a–e; Table 1). Moreover, we observed a significant Pearson’s product-moment correlation $(r)$ among the quality traits *plus* TGW and GY. The GPC exhibited a significant negative correlation with both GSC and GY while a positive correlation with GH. On the other hand, GSC showed a positive correlation with GY and was negatively correlated with GH. Interestingly, TGW was generally neutral for the investigated quality traits (Fig. 1f). The ANOVA revealed that both genotypic and environmental variation was significantly larger than zero with the broad-sense heritability estimates ranging from 0.88 to 0.91 (Table 1 and Table S3a–c), exhibiting the high quality of the phenotypic data. The significant genotypic variation and high broad-sense heritability estimates are imperative for efficient genome-wide association studies (GWAS) and genome-wide prediction of the traits.

**Table 1:** Summary statistics of the investigated wheat grain traits, viz., grain protein content (GPC), grain starch content (GSC), and grain hardness (GH).

Parameter	GPC	GSC	GH
Minimum	10.57	66.56	40.62
Mean	11.66	69.20	50.48
Maximum	14.14	70.58	57.65
Environments	8	3	4
${\sigma }_{G}^{2}$	0.29 ^α^	0.55 ^α^	10.32 ^α^
${\sigma }_{E}^{2}$	1.04 ^α^	1.36 ^α^	0.44 ^α^
${\sigma }_{e}^{2}$	0.24	0.22	5.13
${H}^{2}$	0.91	0.88	0.91

*Environments* number of environments in which the corresponding trait was investigated, ${\sigma }_{G}^{2}$ genotypic variance, ${\sigma }_{E}^{2}$ environmental variance, ${\sigma }_{e}^{2}$ residual variance, α significant at the $P<0.001$.

### Population structure and linkage disequilibrium do not alter significantly by increasing the marker density

In the first step, the whole panel (*n* = 372) was genotyped with the SNP genotyping (35 k and 90 k) arrays *plus* 27 functional candidate-gene markers, which resulted in 26,694 high-quality markers $(p)$ with 10,823 markers having known genetic mapping $(\widehat{p})$ locations (Figs. S2 and S3; Table S4a, b). Secondly, we increased the marker density on a sub-set (half) of the population (*n* = 186; Trost-set)—that represented the complete genotypic diversity space of the full-set—by additionally genotyping with the 135 k array (Fig. S4; Table S4c). In total, the quality criteria imposed on the markers combined from the arrays (35 k, 90 k, and 135 k) *plus* the candidate genes on the trost-set resulted in 47,245 high-quality markers, including 29,359 markers with known genetic mapping $(\widehat{p})$ locations (Fig. S5; Table S4c).

The population structure analyzed via principal component (PC) analysis showed the absence of distinct sub-populations in our full-set panel with the first two PCs explaining only 12.6% of the total variation, most possibly because the panel was composed of recently registered European wheat varieties. However, there was a clear classification among the varieties based on the alleles of the *Rht-D1* locus (Fig. S2b). The PC analysis on the representative trost-set with increased marker density yielded similar results, and the first two PCs explained only 11.1% of the total variance (Fig. S5). Since the trost-set is representative of the genotypes in the full-set, the distribution of panel based on *Rht-D1* marker alleles remained similar (Fig. S5b). Further detailed analyses of the structure of the population have been presented earlier using various marker types and densities^46,47^, and despite increasing the marker density in this study, our results remain consistent with the previous studies.

The linkage disequilibrium (LD; ${r}^{2}$) decayed rapidly by increasing the genetic distances. The LD decay flattened at ~ 5-cM in both full- and trost-set panels, suggesting that—as observed in the PC analyses—increasing marker density above a certain level neither improves population stratification nor LD-decay pattern (Figs. S3a and S6a). The sub-genome-wise allocation of the marker loci followed the expected distribution in both full- and trost-set panels; most of the markers were mapped on the B-genome followed by A- and D-genomes (Figs. S3b and S6b). Nevertheless, as shown in a previous study^47^, it should be noted that an increase in marker density may help in capturing the loci that impart increased genotypic variance and is, therefore, vital for the GWAS.

### GWAS reveal medium- to large-effect loci and putative candidate genes in the full-set of wheat varieties

**Figure 2:**
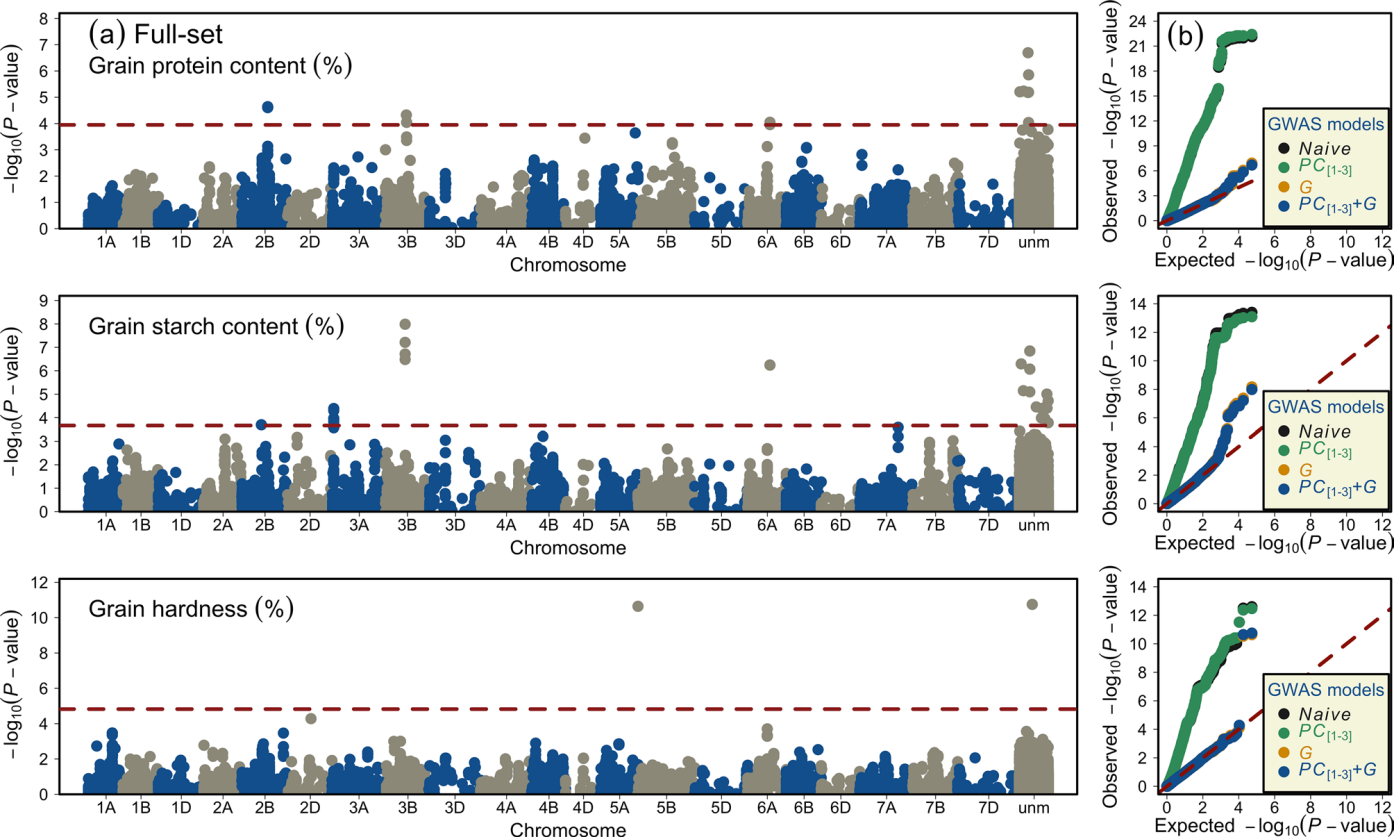
Summary of the genome-wide association studies (GWAS) of investigated traits, viz., grain protein content, grain starch content, and grain hardness in a panel of 372 registered wheat varieties. (**a**) Manhattan plots show the distribution of marker significance $({-log}_{10}P-value)$ along wheat chromosomes. The dashed red line indicates the significance threshold based on false discovery rate (FDR) of $P<0.20$. (**b**) Quantile–quantile plots show the distribution of observed *vs*. expected (red dashed line) ${-log}_{10}(P-value)$. The naïve represents the GWAS without correction for population structure, the *PC*_[1–3]_ represents the GWAS with population structure corrected with the first three principal components (*PC*), the *G* represents the GWAS with familial relatedness corrected with a genomic relationship matrix (*G*), and the *PC*_[1–3]_ + *G* represents the GWAS corrected with both *PC*_[1–3]_ and *G* matrix. Different GWAS models are color coded*,* and the results of *PC*_[1–3]_ + *G* model are displayed in the Manhattan plots for individual traits.

We performed GWAS based on different statistical models and, consistent with the theory, the model correcting for both the population structure and familial relatedness was the most stringent to avoid type-l errors and, therefore, was adopted in this study. The risk of type-ll errors was avoided by observing the distribution of null *vs.* alternative hypotheses in quantile–quantile (qq) plots. Our GWAS revealed the quantitative genetic nature of the studied traits and identified marker-trait associations (MTA) on chromosomes 2B, 3B, and 6A for GPC; 2B, 3A, 3B, and 6A for GSC; and chromosome 5D for GH (Fig. 2; Tables 2 and S5a–c). In total, 15 MTA were detected for GPC, while 29 and two MTA were detected for GSC and GH, respectively. The total genotypic variance (${p}_{G}$) imparted by all MTA for GPC, GSC, and GH amounted to 19.75%, 34.56%, and 14.66%, respectively. Since, 35 MTA were unmapped according to the genetic map used in our study (i.e., based on ITMI mapping population), the chromosome and genetic position of unmapped MTA were retrieved from other published studies^48,49^—this helped to assign 14 more markers to the chromosomes. It should be noted that the chromosomal assignments of the MTA from both the mapping resources generally concur, but the genetic positions differ—this is because the genetic positions were calculated based on different mapping populations. The largest amount of variation was explained by chromosome 2B-QTL (*QGpc.ipk-2B*; ${p}_{G}$ = 11.41%) for GPC; 6A-QTL (*QGsc.ipk-6A*; ${p}_{G}$ = 13.20%) for GSC; and 5D-QTL (*QGh.ipk-5D*; ${p}_{G}$ = 14.91%) for GH (Table 2). Interestingly, the chromosome 6A-QTL, i.e., *QGpc.ipk-6A* and *QGsc.ipk-6A*—represented by markers *AX-94973054* and *Tdurum_contig46828_730* (70.20-cM), respectively—controlled both the GPC and GSC; these two markers imparted 8.37% and 8.21% of the genotypic variance for GPC and 13.06% and 13.20% for GSC, respectively (Table S5a,b).

**Table 2:** Position and genotypic variance of QTL identified in the full set of wheat varieties for grain protein content (*QGpc.ipk*), grain starch content (*QGsc.ipk*), and grain hardness (*QGh.ipk*).

QTL	Chr	Pos. (Mb)*	MTA	Marker	${p}_{G}$
*QGpc.ipk-2B*	2B	658–674	7	*wsnp_BG274584B_Ta_2_3*	11.41
*QGpc.ipk-3B*	3B	731	3	*AX-94457592*	6.04
*QGpc.ipk-6A*	6A	572	2	*AX-94973054*	8.37
*QGsc.ipk-2B*	2B	106	2	*BS00009540_51*	-0.17
*QGsc.ipk-3A*	3A	14–15	5	*CAP11_c6193_232*	5.01
*QGsc.ipk-3B*	3B	674–677	10	*AX-94842052*	10.34
*QGsc.ipk-6A*	6A	572	2	*Tdurum_contig46828_730*	13.20
*QGh.ipk-5D*	5D	4	2	*AX-94991433*	14.91

*Physical position of the markers based on wheat RefSeq *v*1.1.

*MTA* the number of significant marker-trait associations, *Marker* the name of the marker explaining the largest amount of genotypic variance, ${p}_{G}$ percentage of the genotypic variance explained by the corresponding marker.

### Representative genetic nature and GWAS of the trost-set help confirm the full-set’s QTL with increased intra-QTL marker density

Being the representative of the full-set (Fig S4b), the phenotypic distribution, genotypic variance, and average across-environment Pearson’s product-moment correlation of the trost-set lines mirrored the full-set (Figs. S1a–c and Fig. S7a–c; Tables S6a–c and Table S7a–c). The additional genotyping of the trost-set with the 135 k marker array resulted in high-quality markers (*p* = 47,245) that were ~ 1.77 times more than the full-set’s marker (*p* = 26,694) genotypes (Figs. S2a and S5a). As stated elsewhere, the high-end genotyping array was employed to see (1) the impact of high marker density on the PC and LD analyses, and (2) to confirm the full-set’s QTL with high intra-QTL marker density. From our PC analysis of the trost-set, the increased marker density showed similar results as for the full-set: the two-dimensional scatter plot of the first two PCs—as seen for the full-set (Fig. S2b)—showed the separation of the varieties based on the *Rht-D1* alleles and explained only 11.1% of the total variance (Figs. S5b). Similar to the PC analyses, our LD analyses on the trost-set also showed a similar trend, with LD decaying at ~ 5-cM (Fig. S6a). The PC and LD analyses on the representative trost-set, nevertheless, show that the full-set’s total genotypic diversity space was adequately covered.

In total, GWAS performed on the trost-set—by keeping the same model parameters, as described for the full-set—identified the MTA on chromosomes 2B, 5A, and 6A for GPC, and 5A and 5D for GH. No MTA could be identified for GSC (Fig. S8; Table S8a–c). The increased marker density in trost-set resulted in the detection of 28 MTA for GPC and four for GH. As expected, the increased marker density helped in capturing the improved total genotypic variance imparted by the complete set of MTA that amounted to 56.16% and 39.21% for GPC and GH, respectively; substantially larger than that explained by the full-set’s complete MTA, i.e., 19.75% and 14.66% for GPC and GH, respectively. The largest amount of variation was explained for GPC by the QTL on chromosome 6A (*QGpc.ipk-6A*; ${p}_{G}$ = 23.42%), and GH on chromosome 5D (*QGh.ipk-5D*; ${p}_{G}$ = 16.75%).

### *QGpc.ipk-6A* shows opposite allelic nature for grain protein and grain starch content in wheat

**Figure 3:**
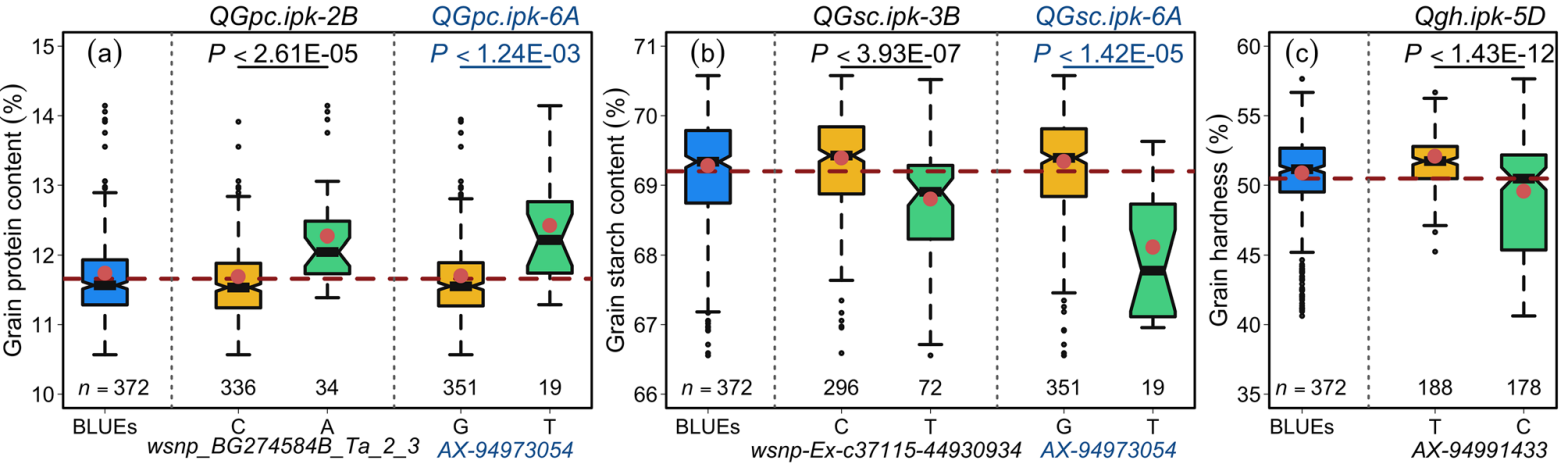
Allele-wise phenotypic distribution of the most significant markers associated with (**a**) grain protein content (%), (**b**) grain starch content (%), and (**c**) grain hardness (%). The alleles of marker *AX-94973054* (blue color) on chromosome 6A display opposite effects for grain protein and starch content.

**Figure 4:**
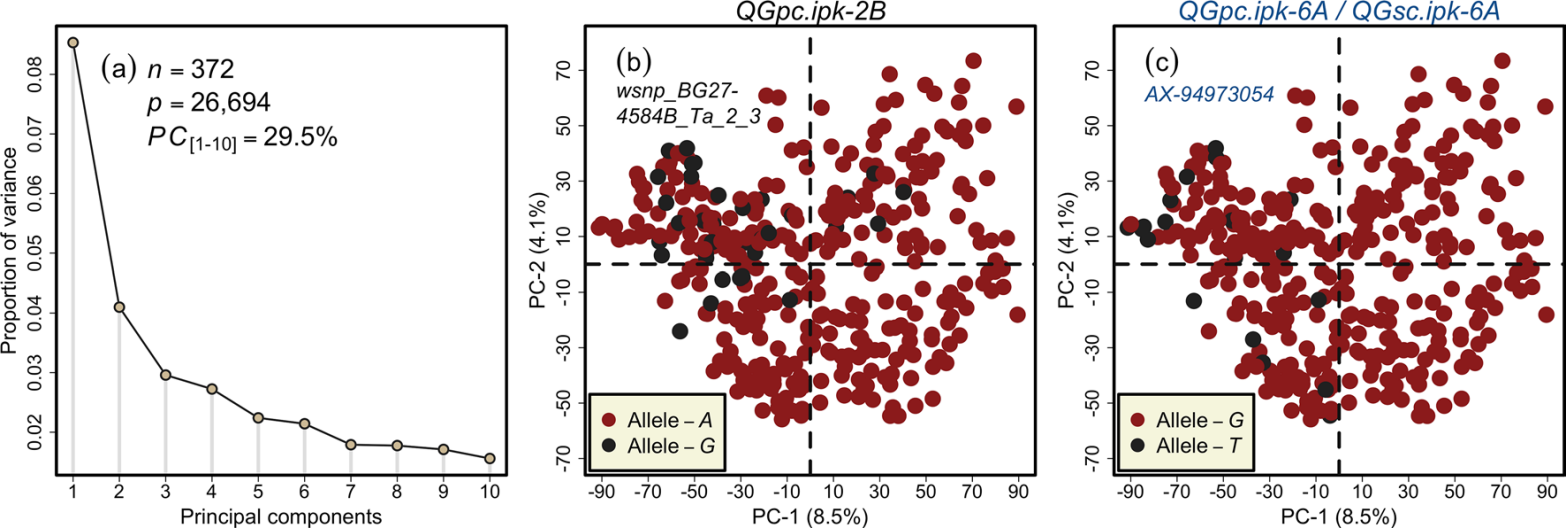
Principal component (PC) analysis of wheat varieties based on the high-quality marker loci combined from the 35 k and 90 k single nucleotide polymorphism arrays *plus* 27 candidate-genes markers. (**a**) The scree plot shows the first ten *PCs* and their corresponding proportion of variance. (**b**) The two-dimensional scatterplot shows the absence of pronounced sub-clustering among the investigated wheat varieties. The varieties are highlighted based on GPC associated marker *wsnp_BG274584B_Ta_2_3* alleles representing the QTL *QGpc.ipk-2B* on chromosome 2B, (**c**) the two-dimensional scatterplot shows the absence of pronounced sub-clustering among the investigated wheat varieties. The varieties are highlighted based on GPC/GSC associated marker *AX-94973054* alleles representing the QTL *QGpc.ipk-6A/QGsc.ipk-6A* on chromosome 6A. *n* and *p* denote the number of varieties and the number of high-quality markers used in the analyses, respectively.

Two markers on chromosome 6A, viz., *AX-94973054* and *Tdurum_contig46828_730* were significant for both GPC and GSC. For further analyses, we selected the marker *AX-94973054* as the representative SNP of the 6A-QTL since it imparted more genotypic variance as compared to *Tdurum_contig46828_730* (Table S5a,b). The box-and-whisker plots of *AX-94973054* marker alleles revealed an opposite allelic effect for GPC and GSC: *AX-94973054-T* increased the GPC but decreased GSC (Fig. 3a,b). Two more loci for GPC were detected on chromosomes 2B (with a total of 10 markers, including the most significant marker *wsnp_BG274584B_Ta_2_3*), and 3B with three markers (Table S5a). However, none of those markers showed an effect on GSC. For both loci, i.e., *QGpc.ipk-2B* and *QGpc.ipk-6A*, a clustering of varieties were observed in the PCA reflecting a relatedness of the high protein varieties (Fig. 4a–c; Table S1). GPC-increasing alleles were rare with a frequency of 5.1%, 7.0%, and 9.1% for *AX-94973054-T, AX-94457592-T*, and *wsnp_BG274584B_Ta_2_3-G*, respectively. Moreover, the allelic distribution in the varieties showed that GPC increasing alleles were enriched in the top third of varieties. Three varieties (i.e., Runal, Lona, and Mewa) carried all three GPC increasing alleles of *QGpc.ipk-2B, QGpc.ipk-3B,* and *QGpc.ipk-6A*—Runal and Lona were the best GPC perfomers (Table S1a).

### Mapping of the markers on the physical map helps to narrow-down the QTL intervals

In total, 18 unique high-confidence and one low-confidence gene-IDs were obtained by BLASTing the sequences of MTA to the corresponding chromosomes of the reference sequence (RefSeq *v*1.1) of the wheat genome (Table S5a–c). For each trait, the MTA explaining the maximum genotypic variance (*QGpc.ipk-2B* for GPC, *QGsc.ipk-6A* for GSC, and *QGh.ipk-5D* for GH) were further investigated.

For *QGpc.ipk-2B*, seven markers (including *wsnp_BG274584B_Ta_2_3*) hit two different genes within an interval of ~ 50-kb (658-Mb) that encode a basic helix-loop-helix (bHLH) transcription factor (*TraesCS2B02G463800*) and a receptor-like protein kinase (*TraesCS2B02G464000*) (Table 3, Table S5a). Three additional MTA (i.e., *BobWhite_c47573_560*, *BS00046166_51*, and *AX-94507002*) clustered ~ 16-Mb downstream (674-Mb) representing a second potential locus, but as they explained only up to 0.89% of the genotypic variance, they were not investigated further.

**Table 3:** Functional annotation, haplotype structure, and nucleotide diversity within the haplotype block of *QGpc.ipk-2B.*

Gene ID	Functional annotation	Length	H	SS	NS	Pi
***TraesCS2B02G463800***	Basic helix-loop-helix (BHLH) transcription factor	3,704	4	2	2	1.60E-03
*TraesCS2B02G463900*	Tudor/PWWP/MBT superfamily protein	6,312	3	4	3	1.85E-03
***TraesCS2B02G464000***	Receptor-like protein kinase	2,439	2	21	10	7.57E-03
*TraesCS2B02G464100*	Kinase, putative	4,969	4	88	243	1.10E-01
*TraesCS2B02G464200*	LEAFY-like protein	3,265	2	0	1	1.10E-03
*TraesCS2B02G464300*	50S ribosomal protein L11	1944	4	0	0	1.18E-03
*TraesCS2B02G464400*	Seed specific protein Bn15D1B	3,597	4	2	3	3.92E-02
*TraesCS2B02G464500*	Single hybrid motif superfamily protein	7,969	3	5	19	1.40E-03

The genes indicated in bold were hit directly by the markers.

*Gene ID* gene identifier retrieved from wheat RefSeq *v*1.1, *H* the number of haplotypes, *SS* the number of synonymous substitutions, *NS* the number of non-synonymous substitutions, *Pi* the nucleotide diversity.

The QTL responsible for both GPC and GSC on chromosome 6A was represented by the markers *AX-94973054* and *Tdurum_contig46828_730*. These markers were located ~ 75-kb apart and BLASTed on genes *TraesCS6A01G338300* and *TraesCS6A02G338600* that encoded a kinase family protein and an aminotransferase-related family protein, respectively (Table 4, Table S5a,b).

**Table 4:** Functional annotation, haplotype structure, and nucleotide diversity within the haplotype block of *QGpc.ipk-6A*.

Gene ID	Functional annotation	Length	H	SS	NS	Pi
*TraesCS6A02G336900*	FRIGIDA interacting protein 1	5,854	3	0	0	1.90E-03
*TraesCS6A02G337000*	Pentatricopeptide repeat-containing protein	4,947	2	1(0)^α^	0	1.60E-03
*TraesCS6A02G337100*	70 kDa heat shock protein	5,635	2	1(0)^α^	0	1.10E-03
*TraesCS6A02G337200*	Chalcone–flavonone isomerase	4,034	2	0	0	2.90E-04
*TraesCS6A02G337300*	Endoglucanase	3,884	3	1	1	2.52E-03
*TraesCS6A02G337400*	cotton fiber protein	1,047	2	0	1	3.70E-04
*TraesCS6A02G337500*	50S ribosomal protein L14	546	2	0	0	1.60E-03
*TraesCS6A02G337600*	Xyloglucan endotransglucosylase/hydrolase	1,362	2	3	1	1.98E-03
*TraesCS6A02G337700*	MTD1	3,903	2	2	0	4.80E-04
*TraesCS6A02G337800*	Subtilisin-like protease	2,740	2	1	4	9.80E-04
*TraesCS6A02G337900*	Subtilisin-like protease	2,341	3	3	4^β^	1.45E-03
*TraesCS6A02G338000*	BES1/BZR1 homolog 1	3,052	2	1	3	2.02E-03
*TraesCS6A02G338200*	Kinase family protein	5,167	2	2	3	1.32E-03
***TraesCS6A02G338300***	Kinase family protein	4,302	3	10	16^β^	1.06E-02
*TraesCS6A02G338400*	MADS-box transcription factor	768	2	6	6	5.60E-03
*TraesCS6A02G338500*	Subtilisin-like protease	1,132	2	8	14	2.01E-02
***TraesCS6A02G338600***	Aminotransferase-related family protein	3,004	2	6(10)^α^	1	2.43E-03
*TraesCS6A02G338700*	UDP-3-O-acylglucosamine N-acyltransferase	587	3	0	1	9.70E-04
*TraesCS6A02G338800*	DNA (Cytosine-5-)-methyltransferase	6,048	2	2	4	9.20E-04
*TraesCS6A02G338900*	Pentatricopeptide repeat-containing protein	2008	2	3	1	7.70E-04

The genes indicated in bold were hit directly by the markers. α represents when multiple transcripts were annotated, the number of mutations is indicated if different. β indicate genes having a splice region variant compared to the reference sequence.

*Gene ID* gene identifier retrieved from wheat RefSeq *v*1.1, *H* the number of haplotypes *SS* the number of synonymous substitutions, *NS* the number of non-synonymous substitutions, *Pi* and the nucleotide diversity.

The MTA detected for 5D-QTL of GH corresponded to the gene *TraesCS5D02G004300*, that encodes *Puroindoline-b*, providing a positive proof of the efficiency of GWAS to detect true MTA.

### Characterization of the physical regions of *QGpc.ipk-2B* and *QGpc.ipk-6A* revealed potential candidate genes for grain protein content in wheat

To analyze the two physical regions, we used a similar strategy, as described in Muqaddasi et al.^50^. More specifically, ~ 2-Mb (1-Mb upstream and downstream) wheat reference genomic sequence around the most significant markers was retrieved to characterize the QTL physical region.

**Figure 5:**
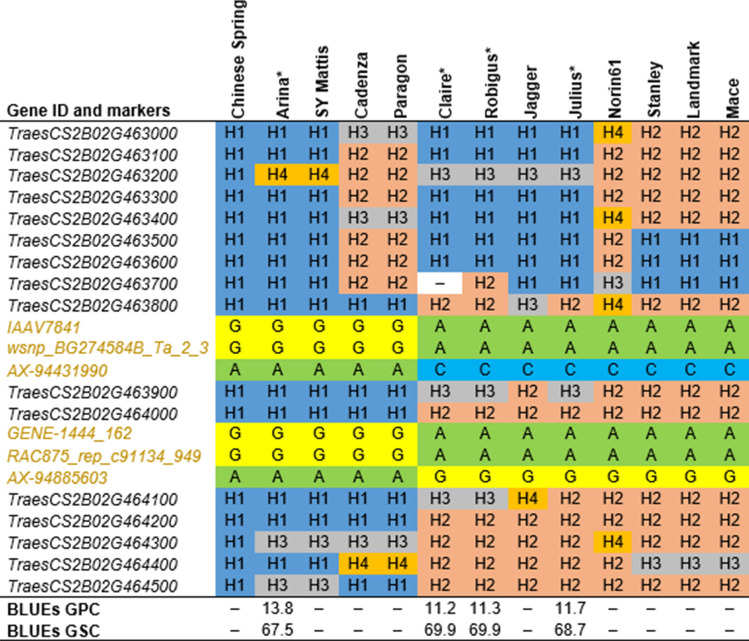
Haplotype structure across 12 wheat varieties obtained from *The 10* + *Wheat Genome Project* in the ~ 2-Mb sequence window around the most significant marker of *QGpc.ipk-2B*. The varieties included in the GWAS analyses are indicated with an asterisk, and their best linear unbiased estimations (BLUEs) for grain protein content (GPC) and grain starch content (GSC) are given at the bottom. The marker names (highlighted) are located below the gene IDs from the wheat RefSeq *v*1.1 that they hit. For each gene, the haplotype structure is indicated in reference to Chinese Spring and then numbered according to their frequency across the 12 varieties. An – indicates the missing data. The allelic information of a given variety is provided for the markers.

The physical region of *QGpc.ipk-2B* (656.79–658.85-Mb) harbored 16 high-confidence genes (Table S9a). The functional annotation of these genes revealed transcription factors, P-loop NTPases, and protein kinases. To narrow-down to putative candidate genes for GPC, the haplotype structure and the nucleotide diversity of 26 genes were investigated across 12 wheat varieties sequenced within the framework of *The 10* + *Wheat Genome Project* (https://www.10wheatgenomes.com/). Despite four genes being highly conserved across the 12 analyzed varieties, the nucleotide diversity within the region was relatively high. In total, up to four haplotypes were identified (Table 3): four varieties (Arina, Cadenza, Paragon, and SY-Mattis) shared the same haplotype as Chinese Spring over an interval of eight genes (from *TraesCS2B02G463800* to *TraesCS2B02G464500*)—a genomic region that harbored genes hit directly by the markers (Fig. 5). These varieties harbored the allele *wsnp_BG274584B_Ta_2_3-G* that increased the GPC. For example, Arina, a variety present both in our GWAS panel and sequenced within the pan-genome framework (*The 10* + *Wheat Genome Project*), showed a high GPC. Two genes showed a high number of substitutions: the receptor-like protein kinase (*TraesCS2B02G464000*; hit by the marker *AX-158547228*) and the neighboring gene *TraesCS2B02G464100* coding for a putative kinase. The latter displayed a modification in its leader sequence (with a potential alternative start codon 42 nucleotides upstream) and a large number of coding and non-coding substitutions in six varieties from *The 10* + *Wheat Genome Project*.

**Figure 6:**
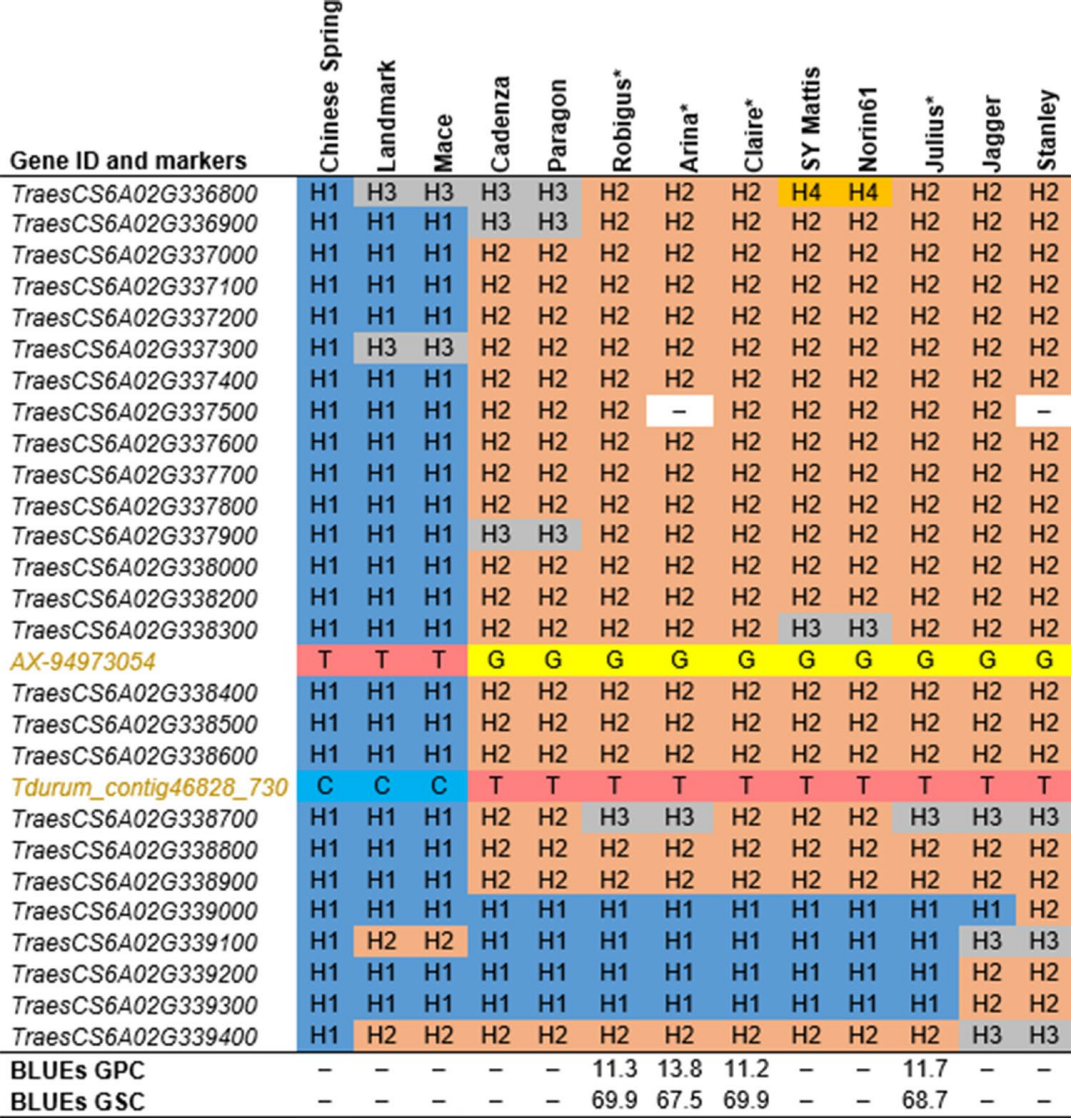
Haplotype structure across 12 wheat varieties obtained from *The 10* + *Wheat Genome Project* in the ~ 2-Mb sequence window around the most significant marker of *QGpc.ipk-6A*. The varieties included in the GWAS analysis are indicated with an asterisk, and their best linear unbiased estimations (BLUEs) for grain protein content (GPC) and grain starch content (GSC) are given at the bottom. The marker names (highlighted) are located below the gene IDs from the wheat RefSeq *v*1.1 that they hit. For each gene, the haplotype structure is indicated in reference to Chinese Spring and then numbered according to their frequency across the 12 varieties. An – indicates the missing data. The allelic information of a given variety is provided for the markers.

The *QGpc.ipk-6A* physical region (570.85–572.93-Mb) harbored 26 high-confidence genes (Table S9b), and the functional annotation of the genes indicated the presence of subtilisin-like proteases, proteins involved in the degradation of cellulose and cell-walls, kinases, and also transcription factors. A block of 20 genes (from *TraesCS6A02G336900* to *TraesCS6A02G338900*) formed two major haplotypes with two varieties (Landmark and Mace), shared the same haplotype as Chinese Spring, and harbored the marker allele *AX-94973054-T*: an allele which increased GPC but decreased GSC (Fig. 6). Interestingly, applying the gene models from Chinese Spring over the QTL interval revealed a high number of substitutions, including up to 54 synonymous (depending on the splice variants considered) and at least 61 non-synonymous mutations (Table 4). In the QTL region, two genes, in particular, were affected by large-effect mutations compared to Chinese Spring: a 558-bp deletion in the 3′ region of the subtilisin-like protease (*TraesCS6A02G337900*) and a splice region variant overlapping with the U-box domain of the kinase family protein *TraesCS6A02G338300*.

### The accuracy of genome-wide prediction for grain quality traits suggest the efficient use of genome-wide selection in wheat breeding programs

**Figure 7:**
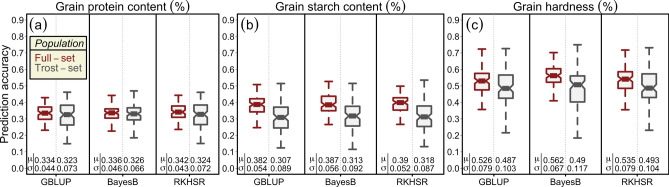
Accuracy of the genome-wide prediction for (**a**) grain protein content (%), (**b**) grain starch content (%), and (**c**) grain hardness (%) based on three different genomic selection models viz., genomic best linear unbiased prediction (GBLUP), BayesB and reproducing kernel Hilbert space regression (RKHSR) evaluated through 100 random runs of fivefold cross-validation cycles. $\mu$ and $\sigma$ denote the mean prediction accuracy and standard deviation of the corresponding model, respectively.

The mean prediction accuracies resulting from the five-fold cross-validation scenario of grain quality traits produced similar results across all three tested model scenarios, i.e., the GBLUP model that accounted for the main additive effects of markers assuming equal variances, BayesB by assuming unequal marker variances, and RKHSR that accounted for both additive and the epistatic interaction among the loci (Fig. 7a–c). Since the size of the training population and marker density are known to affect the outcomes of prediction accuracy, different scenarios were studied by employing both full- and trost-set panels with varying marker densities across the whole genome. The size of the training population seems to be the primary driver of high mean prediction accuracies: prediction accuracies were consistently higher in full-set variety panel as compared to the trost-set. Moreover, the standard deviation was also considerably higher in the trost-set as compared to the full-set (Fig. 7a–c). Nevertheless, it is worth noting that, consistent with the theory, BayesB outperformed both GBLUP and RKHSR for the GH: GH is primarily controlled by a single large-effect *Ha* locus. For GPC and GSC, however, RKHSR slightly outperformed both GBLUP and BayesB, suggesting that epistatic interaction may be prevalent for these traits.

## Discussion

### The use of a diverse variety panel revealed the opposite genetic nature of wheat grain protein *vs.* starch content and yield

Improvement of grain quality parameters is considered as one of the top priorities in applied wheat breeding. While improving grain yield (GY), breeders—especially in the later cycles of breeding programs—pay close attention to the baking and milling quality of wheat grain. Along with biotic and abiotic stress resistance, GY and quality parameters form the basis of the success of an experimental line in the wheat market. Most of the grain quality parameters are heavily influenced by environment, crop management, and soil fertility practices. Nonetheless, there exists a level of variation among wheat lines that points to the underlying genetic factors controlling the quality traits. Exploiting the genetic variation to improve a given trait is a sustainable agricultural practice. We investigated—over several environments—a set of 372 recently developed wheat varieties registered for the European market for three important grain quality traits viz., grain protein content (GPC), grain starch content (GSC), and grain hardness (GH).

The existence of a large and significant genetic variation for GPC, GSC, and GH in the studied wheat varieties reflects the genotypic diversity covering all wheat classes. The large genotypic variance also translated into high broad-sense heritability estimates, pointing to trait-underlying genetic factors. GPC is a major component defining the quality of a wheat variety while GH influences endosperm texture^51^. A high positive correlation between GPC and GH was previously reported^52^ and the physicochemical mechanism connecting puroindolines and the starch-protein interaction have been attributed to an interaction of *PinA* and gliadins influencing the interaction of prolamins with starch granules^53^. Moreover, we observed a significant negative correlation of GPC *vs*. GSC and GY: a phenomenon that was previously reported to be due to the genetic factors^54,55^. To improve both traits simultaneously, breeding-oriented strategies such as multi-trait genomic selection were applied^43^. In addition, genetic modification approaches were also suggested, e.g., the ectopic expression of a barley sucrose transporter in the so-called HOSUT wheat lines resulted in an increased GPC and GY^56,57^. However, genetically modified varieties are not accepted in Europe. Although—as noted earlier—GPC can be influenced by agronomic practices, our goal in this study was to identify the genetic factors determining if a variety is a high or low GPC variety. The QTL described in this manuscript mainly reflect the differences between the different variety types regarding GPC.

### *QGpc.ipk-2B* and *QGpc.ipk-6A* influence grain protein content in European winter wheat varieties

Two QTL regions on chromosomes 2B and 6A were detected in the full-set of varieties, and were confirmed in the trost-set (186 varieties representing the genotypic diversity space of all 372 full-set varieties) with increased marker density. While *QGpc.ipk-2B* only influenced GPC, the 6A-QTL controlled both GPC (*QGpc.ipk-6A*) and GSC (*QGsc.ipk-6A*) with opposite allelic effects. Most of the high GPC varieties carried only the GPC-increasing allele of one locus, indicating that these loci may be present in the germplasm of different breeding programs. In both cases, however, a clustering of the alleles for high GPC was observed in the PCA, highlighting the relatedness of the high GPC varieties. A number of high-GPC varieties, nevertheless, harbored neither of both loci: this may be explained by the relatively low genotypic variances of 8.4% for *QGpc.ipk-6A* and 11.4% for *QGpc.ipk-2B*. This also suggests that additional factors are in place which could not be detected in this study. The most significant loci for GSC were located on chromosomes 3A, 3B, and 6A that explained genotypic variances of 5.0%, 10.3%, and 13.2%, respectively. The detection of a QTL for GH harboring the known *Puroindoline-b* gene on chromosome 5D confirmed the appropriateness of our approach and, therefore, can be regarded as proof of concept.

A meaningful comparison to other studies based on genetic maps is difficult due to the use of different reference populations and marker types/data sets. A more precise comparison is, however, possible by comparing the physical positions of the significant markers; for this strategy, the knowledge about the marker sequences is a prerequisite. Jernigan et al.^11^ reported a QTL for flour protein on chromosome 6A in an association mapping panel of soft white winter wheat cultivars in the U.S. Pacific Northwest. Their reported significant marker *IWB31459* is located at 609.4-Mb while our QTL on chromosome 6A located at 572-Mb (Table 2). Likewise, a GWAS study in durum wheat^17^ reported a GPC QTL on chromosome 3B based on marker *IWB13886*. The physical location of this marker is 725.7-Mb which is relatively close to the 731-Mb of our GPC-QTL (Table 2). Two GPC QTL reported in the same study on chromosome 2B were far from 2B-QTL reported in this study.

### The physical structure of *QGpc.ipk-6A* and *QGpc.ipk-2B*, nucleotide diversity, and candidate gene identification

As previously used to investigate the genetic basis of total spikelet number^50^ and suggested by Borril et al.^58^, the availability of high-quality assemblies of cultivars makes it possible to explore the physical regions associated with the MTA. We analyzed the haplotype structure and the nucleotide diversity of high-confidence genes annotated in an interval of 2-Mb around the most significant markers of *QGpc.ipk-2B* and *QGpc.ipk-6A*. In total, 12 varieties sequenced within the frame of the wheat pan-genome were studied to identify potential candidate genes for GPC.

Our analysis of *QGpc.ipk-6A* revealed that, within a haplotype block of 20 genes, there exists a cluster of genes coding for protein kinases and subtilisin-like proteases. The varieties having the allele *AX-94973054-G* for low GPC and high GSC also had large-effect mutations for the subtilisin-like protease *TraesCS6A02G337900* and the kinase family protein *TraesCS6A02G338300*. The processes involved in the development of wheat grain are certainly multifaceted; however, regulation of vegetative organ senescence appears to be one of the main factors^59^. Subtilisin-like proteases were shown to be induced by senescence, redirecting nutrients from leaves during grain filling in wheat^60^. A similar mechanism was identified for the QTL *GPC-B1* located on chromosome 6B that encoded a NAC transcription factor (NAM-B1) regulating leaf senescence and increasing grain protein, iron, and zinc content^24,25^. Nevertheless, it is important to note that the QTL identified here, i.e., *QGpc.ipk-6A*, is not the homeolog of *GPC-B1* (physical location ~ 77-Mb).

The highly conserved protein kinases are important regulatory components in plant cells. They are involved in many pathways, including hormonal, nutrient, and cell-cycle signaling^61^. Interestingly, the two genes most affected by substitutions in the QTL region *QGpc-ipk-2B* code for a receptor-like kinase and a putative kinase, the latter having probably its leader sequence modified. Therefore, non-functional or differentially regulated alleles at those two loci could be related to low protein content in grain. However, it should be noted that, although the homologous physical regions can be compared across 12 cultivars, the gene annotation is, for the moment, only based on the reference sequence.

### The prospects of predictive breeding for quality traits in applied wheat breeding programs

For GWAS, we used state-of-the-art high-density SNP arrays and multi-environment robust phenotypic data. However, the genotypic variance imparted by total MTA amounted to 19.75%, 34.56%, and 14.66% for GPC, GSC, and GH, respectively. A large amount of unexplained genotypic variance can be ascribed to many small-effect loci and, therefore, extremely complex genetic architecture of the investigated traits. Genome-wide prediction—a method that is used to predict the total genetic value of a trait based on all maker loci irrespective of their effect size—therefore, becomes a method of choice to improve the genetic gain^41^. Selection decisions based on genomic prediction can be robust and may result in higher genetic gain per unit time in comparison to both GWAS when no large-effect QTL are identified, and phenotypic selection. For example, recently Battenfield et al.^62^ reported that genetic gain by employing genomic selection were up to 2.7 times higher than phenotypic selection. Also, the cost of genotyping ~ 10,000 wheat lines was equivalent to the phenotypic evaluation of 2,000 lines. This suggests that, if the genomic prediction accuracy is high, (1) the quality traits can be predicted relatively early in breeding programs to make informed breeding decisions, and (2) genetic gains can be improved per unit of time and capital investment without having to wait till the latter cycles where only the high GY candidates are grown. Based on three different models, our genome-wide prediction accuracy results suggest that genetic gains can be improved with high confidence. Our results are in line with the recent reports, where similar genome-wide prediction accuracies for quality traits were reported on wheat panels comprising of different genetic natures^12,20,42,43,63^. This warrants the efficient use of genomic selection in elite breeding programs to predict the total genetic merit of individuals.

## Conclusion

Coupled with a diverse genotype panel, robust phenotyping data collected from several field locations, and dense molecular markers, the use of modern genomic tools such as the reference sequence and the pan-genome of wheat allowed the detection of two QTL for GPC located on chromosomes 2B and 6A. The physical regions underlying these QTL were studied in detail in 12 sequenced wheat varieties. Putative candidate genes involved (1) protein kinase and kinase family proteins with potential regulatory functions, (2) a subtilisin-like protease that may be involved in the regulation of senescence, (3) transcription factors, and (4) an aminotransferase-related family protein. The confirmation of candidate gene/s will require further functional characterization via genome editing or TILLING approaches. The results of genome-wide prediction show promising prospects in applied breeding to improve genetic gains for quality traits.

## Material and methods

### Field trials, collection, and analyses of the phenotypic data

An elite European winter wheat panel (GABI) comprising 372 varieties (358 winter type; 14 spring type) was evaluated for three major quality traits viz., grain protein content (GPC), grain starch content (GSC), and grain hardness (GH). The phenotypic data of the quality traits were gathered from three to eight environments. Each environment was considered a location-by-year combination. The field trials were conducted in an alpha lattice design with two replications per environment. More details about the field trials, agronomic practices, climatic conditions, and calculation of the adjusted entry means per environment are described in Zanke et al.^64^. The wheat quality parameters were evaluated using a standard near-infrared approach. The phenotypic measurements were carried out by the collaborating seed companies by using sample volumes of 400 g grains per harvested field plot and an OmegAnalyzer G (Bruins Instruments) applying wavelengths of 730–1100 nm.

The consistency among the individual trait values was investigated by primarily drawing environment-specific adjusted mean values as box-and-whisker plots. Moreover, to check the general performance of a given trait across environments, we calculated the average correlation by performing Fisher's $z$ transformation^65^ as follows:

Firstly, we calculated bivariate Pearson's product-moment correlation $(r)$ as:

1 $$r=\frac{cov\left(x,y\right)}{{\sigma }_{x}{\sigma }_{y}}=\frac{{\sum }_{i=1}^{n}{(x}_{i}-\stackrel{-}{x})({y}_{i}-\stackrel{-}{y})}{\sqrt{{\sum }_{i=1}^{n}{{(x}_{i}-\stackrel{-}{x})}^{2}}\sqrt{{\sum }_{i=1}^{n}{\left({y}_{i}-\stackrel{-}{y}\right)}^{2}}}$$

Secondly, each bivariate correlation coefficient was transformed into Fisher's $z$ as:

2 $$z=\frac{1}{2}ln\left(\frac{1+r}{1-r}\right)$$

Thirdly, the mean $z$ was back-transformed to $\stackrel{-}{r}$ as:

3 $$\stackrel{-}{r}=\frac{{e}^{\left(2z\right)}-1}{{e}^{\left(2z\right)}+1}$$

The above transformation is shown to provide a less-biased estimate of the average correlation as compared to the average correlation that is estimated based only on the mean of correlation values^66^.

To calculate across-environment individual variance components of the genotype, environment, and the residuals, the following linear mixed effect model was used by assuming all effects except the intercept as random:

4 $${y}_{ij}=\mu +{G}_{i}+{E}_{j}+{e}_{ij}$$

where, ${y}_{ij}$ is the phenotypic value (adjusted mean value of a given trait in each environment) of the ${i}$th genotype in the ${j}$th environment, $\mu$ is the common intercept term, ${G}_{i}$ is the effect of the ${i}$th genotype, ${E}_{j}$ is the effect of the ${j}$th environment, and ${e}_{ij}$ is the corresponding residual term. The broad-sense heritability $({H}^{2})$ was calculated as:

5 $${H}^{2}=\frac{{\sigma }_{G}^{2}}{{\sigma }_{G}^{2}+\left(\frac{{\sigma }_{e}^{2}}{nE}\right)}$$

where ${\sigma }_{G}^{2}$ and ${\sigma }_{e}^{2}$ denote the variance components of the genotype and residuals, respectively, and $nE$ represent the number of environments. The best linear unbiased estimations (BLUEs) across environments were calculated by assuming the effects of the intercept and genotype as fixed in Eq. 4. Moreover, the genetic correlations among all the traits were calculated based on their BLUEs computed across environments, as described in Eq. (1).

### Analyses of the genotypic data, linkage disequilibrium, and population structure

The whole wheat panel (*n* = 372) was genotyped with state-of-the-art 35 k Affymetrix and 90 k iSELECT single nucleotide polymorphism (SNP) arrays that generated 35,143 and 81,587 SNP markers (*p*), respectively. Furthermore, the whole wheat panel was genotyped with 27 candidate-gene markers, as described in Schulthess et al.^45^. The genetic mapping positions were adopted from the International Triticeae Mapping Initiative (ITMI) map, as described in Sorrels et al.^67^. In total, 35 k and 90 k arrays resulted in 13,344 and 11,676 mapped marker loci. The SNP markers from both arrays *plus* the scores of the candidate gene markers were combined (resulting in an $n\times p$ matrix of 372 $\times$ 116,757) and subjected to the quality check. The quality criteria were implemented to remove the markers with a minimum of 0.05 minor allele frequency and > 5% missing or heterozygous calls; the remaining missing or heterozygous calls were imputed with the mean value of both alleles.

The extent of linkage disequilibrium (LD; the non-random association of alleles at two or more loci) decay over genetic distance greatly impacts the outcome of GWAS and genomic prediction. The genome-wide LD was measured based on the squared correlation $({r}^{2})$ method^68^ among all the mapped quality markers as:

6 $${r}^{2}=\frac{{\left({p}_{ab}-{p}_{a}{p}_{b}\right)}^{2}}{{p}_{a}\left(1-{p}_{a}\right){p}_{b}\left(1-{p}_{b}\right)}$$

where, ${p}_{a}$ and $\left(1-{p}_{a}\right)$ denote the frequency of two alleles (*a* and *-a*) at locus 1, ${p}_{b}$ and $\left(1-{p}_{b}\right)$ denote the frequency of two alleles (*b* and *-b*) at locus-2, and ${p}_{ab}$ is the frequency of haplotypes harboring alleles *a* and *b* at the loci 1 and 2, respectively. The LD values among the adjacent markers were plotted against the genetic distance in the form of boxplots, as described in Muqaddasi et al.^47^.

The population structure was assessed by principal component (PC) analysis via singular value decomposition. The scree plot (depicting the proportion of variance explained by the first ten PCs) and two-dimensional scatter plots (showing the grouping of the varieties based on the first two eigenvectors) were plotted to show the variance present in the studied wheat panel.

### The selection criterion of the representative sub-set, high-density genotyping, and genetic analyses

Based on the hypothesis that improved marker density across the genome helps improve the outcome of genetic analyses, we selected a sub-set (*n* = 186, named as trost-set) of varieties representing the full-set of varieties (*n* = 372). The exercise of increased marker density in the representative set was, in particular, executed to (1) check if increased marker density substantially affects the outcome of PC and LD analyses, (2) confirm the QTL detected in the full-set of varieties, (3) increase the intra-QTL marker density to identify the trait underlying candidate genes, and (4) estimate the impact of increased marker density on the genome-wide prediction accuracy of a given trait as opposed to the size of training population. The trost-set selection criterion was based on the varieties covering the complete genotypic diversity space of the full-set revealed in the PC analyses.

After sub-panel’s selection, we genotyped the trost-set with a high-end 135 k Affymetrix SNP array (https://www.traitgenetics.com). In total, the 135 k array yielded 136,780 SNP markers; 41,171 markers were mapped according to the ITMI mapping resources. We combined the trost-set’s 135 k markers with the full-set’s 35 k, 90 k, and candidate-gene markers that resulted in an $n\times p$ matrix of 186 $\times$ 253,537. To obtain high-quality makers, we implemented the filtering, as mentioned above for the full set. On the quality trost-set markers, we performed the PC and LD analyses, as described above.

### Genome-wide association studies

The whole panel was evaluated for the presence of the trait-linked markers via genome-wide association studies (GWAS). Let $n$ be the number of wheat lines and $p$ the predictor markers. A standard linear mixed linear model was employed as:

7 $$y=1\mu +X\beta +Pv+Zu+e$$

where $y$ is the column vector of BLUEs of each genotype calculated in Eq. 4, $\mu$ is the common intercept, $\beta ,v, u$, and $e$ are the vectors of markers, population structure (principal components), polygenic background, and the error effects, respectively; $X, P,$ and $Z$ are the corresponding design matrices. In the model, $\mu , \beta ,$ and $v$ were assumed to be fixed while $u$ and $e$ as random with $u\sim N\left(0,G{\sigma }_{a}^{2}\right)$ and $e\sim N(0,I{\sigma }_{e}^{2})$. The $n\times n$ variance-covariance additive genomic relationship matrix $(G)$ was calculated from an $n\times p$ matrix $W=({w}_{ik})$ of marker genotypes (being 0, 1, or 2) as:

8 $$G=\frac{\sum_{k=1}^{p}\left({w}_{ik}-2{p}_{k}\right)\left({w}_{jk}-2{p}_{k}\right)}{2\sum_{k=1}^{p}{p}_{k}\left(1-{p}_{k}\right)}$$

where ${w}_{ik}$ and ${w}_{jk}$ are the profiles of the ${k}$th marker for the ${i}$th and ${j}$th variety, respectively; ${p}_{k}$ is the estimated frequency of one allele in ${k}$th marker, as described by VanRaden^69^. Since population stratification and familial relatedness can severely impact the power to detect the true marker-trait associations (MTA) in GWAS, different methods were used to correct for population stratification and relatedness viz., (1) multiple linear regression (*naïve*), (2) correction of population structure by the first three principal components (*PC*_[1–3]_), (3) correction of familial relatedness via genomic relationship matrix *G*, and (4) correction of both population structure and familial relatedness by *PC*_[1–3]_ and *G*. It is expected that correcting for both *PCs* and *G* in the model enhances the detection accuracy of MTA in GWAS. The models described above were compared by plotting expected *vs.* observed ${-log}_{10}(P)$ values in the form of a quantile–quantile (qq) plot. The best model was determined by checking how well the observed ${-log}_{10}(P)$ values aligned with the expected.

To declare the MTA, a liberal false discovery rate (FDR) to account for multiple testing was applied at *P* < 0.20^70^. As described by Utz et al.^71^, the genotypic variance (${p}_{G}$) explained by all QTL was determined as:

9 $${p}_{G}=\left(\frac{{R}_{adj}^{2}}{{H}^{2}}\right)\times 100$$

where ${R}_{adj}^{2}$ was calculated as ${R}_{adj}^{2}={R}^{2}-\left(\frac{{z}^{^{\prime}}}{N-{z}^{^{\prime}}-1}\right)\left(1-{R}^{2}\right)$ by fitting the MTA $({z}^{^{\prime}})$ in the order of their descending *P*-values in a multiple linear regression model; ${R}^{2}, N,$ and ${H}^{2}$ denote the regression coefficient, number of observations, and the broad-sense heritability calculated in Eq. 5, respectively. The ${p}_{G}$ explained by the individual MTA was accordingly calculated from their sum of squares.

### Identification of candidate genes and analyses of the haplotypes based on the wheat varieties sequenced within *The 10* + *Wheat Genome Project*

The sequences of the significant markers (MTA) were first BLASTed on the corresponding chromosomes of the reference sequence of the wheat genome to retrieve the gene identifiers and their corresponding functional annotations. Furthermore, we recovered the sequences of high confidence genes and their annotated functional descriptions present within a window of 2-Mb (1-Mb upstream and downstream) from the most significant markers for GPC on chromosomes 2B and 6A (*QGpc.ipk-2B* and *QGpc.ipk-6A*). Geneious Prime 2020 (https://www.geneious.com) was used for all BLAST searches and sequence alignments.

To narrow down the QTL regions and identify putative candidate genes, we analyzed the QTL haplotype structure and nucleotide diversity by using the genomic resources available from the wheat pan-genome (https://www.10wheatgenomes.com/). For this purpose, 12 out of the 14 sequenced varieties were analyzed; among them, eight are assembled in pseudomolecules while the remaining four are available only as scaffolds. Two varieties were not included in the final analyses: Spelt systematically carried private alleles whereas Lancer had a highly divergent genomic region on chromosome 2B but shared the same haplotype as the majority of varieties in the *QGpc.ipk-6A* region. Three varieties (Arina, Julius, and Robigus) sequenced within the wheat pan-genome framework were also analyzed in our GWAS analyses (both full- and trost-set) while Claire was only included in the full set. All the gene sequences obtained from the reference sequence (Chinese Spring) were BLASTed against the genomes of the 12 varieties using MegaBlast by retrieving the sequences with a 2-kb context to overcome masked regions. For the varieties assembled in pseudomolecules, the respective chromosomes were used, and the best hits were retrieved. For the remaining varieties, six hits per gene were evaluated and the closest sequence was retained—in case of doubt about the homology, the sequence was omitted. The sequences of each gene were then aligned using MAFFT *v*7.450^72,73^, and SNPs present in the coding regions were called. The number of haplotypes and the nucleotide diversity^74^ were analyzed with DnaSP *v*6^75^.

### Genome-wide prediction

To assess the accuracy of genome-wide prediction for grain quality traits, three different genomic selection models viz., genomic best linear unbiased prediction (GBLUP), BayesB, and reproducing kernel Hilbert space regressions (RKHSR) were employed^41,76–78^.

GBLUP is a standard robust parametric procedure which exploits the additive effects of all the loci to predict the total genetic value of the trait under consideration by assuming the equal effect variances of all loci. It involves the regression of the marker genotypes on the phenotypic data in a linear model of the form:

10 $$y=1\mu +X\beta +e$$

where $\mu$ is a common intercept, $X$ is $n\times p$ incidence matrix of marker genotypes,$\beta$ is a $p\times 1$ vector of marker fixed effects, and $e$ is a $n\times 1$ vector of error term with the assumption that $\beta \sim N\left(0, I{\sigma }_{\beta }^{2}\right)$ and $e\sim N(0,I{\sigma }_{e}^{2})$. By setting $g=X\beta$, GBLUP takes the form as:

11 $$y=1\mu +g+e$$

where $g\sim N(0,G{\sigma }_{a}^{2})$ and $G$ was calculated, as described above in Eq. 8.

In reality, the distribution of genetic variances across loci is not equal, i.e., segregating loci show variance while the non-segregating loci show no variance. BayesB model, being of the same form as Eq. 10, utilizes a scaled inverse Chi-squared $({\chi }^{-2})$ distribution on the marker variances. This circumvents the problem of equal variance by assuming a prior distribution ($\pi$; the prior proportion of non-zero effects) that yield a scaled $t$-distribution for marker effects by using both shrinkage and variable selection methods. Following Pérez and de los Campos (2014)^79^, the prior distribution can be modeled as:

12 $$p\left( {\beta_{j} ,\sigma_{\beta }^{2} ,\pi } \right) = \left\{ {\mathop \prod \limits_{k} \left[ {\pi N\left( {\beta_{jk} {|}0,\sigma_{\beta }^{2} } \right) + \left( {1 - \pi } \right)1\left( {\beta_{jk} = 0} \right)} \right]\chi^{ - 2} \left( {\sigma_{{\beta_{jk} }}^{2} {|}df_{\beta } ,S_{\beta } } \right)} \right\}B(\left( {\pi {|}p_{0} ,\pi_{0} } \right) \times G\left( {S_{\beta } {|}r,s} \right)$$

where, $N$ and $B$ denote normal and beta densities; $\beta$ and ${\sigma }_{\beta }^{2}$ represent the vector of regression coefficients and respective variance. To set the hyper-parameters, we implemented the built-in procedures of BGLR, as described in Pérez and de los Campos (2014) ^79^.

The RKHSR is a semiparametric method that accounts for the additive as well as epistatic interactions among loci. It is of the same form as GBLUP (Eq. 11) with the assumption that $g=K\alpha$, and thus can be represented as:

13 $$y=1\mu +K\alpha +e$$

where, $y,\mu$ and $e$ are the same as described in Eq. 10, and $\alpha$ is the vector of random effects. In RKHSR, $a\sim N(0, K{\sigma }_{\alpha }^{2})$ and $K$ is $n\times n$ symmetric positive-definite matrix and is defined as ${K}_{ij}={e}^{\left(-h\times \frac{{d}_{ij}^{2}}{p}\right)}$ where ${K}_{ij}$ represents the measured relationship between the ${i}$th and ${j}$th variety based on their marker profiles, ${d}_{ij}^{2}$ is the Euclidean distance between the ${i}$th and ${j}$th variety and $h$ is the bandwidth parameter. To determine the optimum $h$, three different values as $h=0.5 \times (1/5, 1, 5)$ were tested in a five-fold cross-validation scenario, and the value representing the highest accuracy was chosen.

We evaluated the accuracy $({r}_{GP})$ of all prediction models by using a five-fold cross-validation scenario. The varieties were randomly divided into five subsets; four of them were used as the training set to estimate the genetic values of the remaining test set. The accuracy of prediction was defined as the Pearson's product-moment correlation between the observed $(y)$ and predicted $(\widehat{y})$ genetic values standardized by the square root of the broad-sense heritability as ${r}_{GP}=\frac{cor\left(y, \widehat{y}\right)}{H}$. Since the cross-validation runs were repeated for 100 cycles, mean and standard deviation values were calculated to show the performance of the individual genomic prediction model to predict the genetic value of the traits. Unless stated otherwise, all calculations were performed in software R^80^ mainly by using packages lme4 and rrBLUP^81,82^.

### Ethical standards

On behalf of all co-authors, the corresponding author states that the work described is original, previously unpublished research. All the authors listed have approved the manuscript.

## Supplementary information

Supplementary file1 (PDF 1567 kb)

Supplementary file2 (XLSX 297 kb)

Supplementary file3 (XLSX 11 kb)

Supplementary file4 (XLSX 12 kb)

Supplementary file5 (XLSX 12 kb)

Supplementary file6 (XLSX 4755 kb)

Supplementary file7 (XLSX 11 kb)

Supplementary file8 (XLSX 12 kb)

Supplementary file9 (XLSX 1630 kb)

Supplementary file10 (XLSX 16 kb)
