# Supplementary material for: Prospects of GWAS and predictive breeding for European winter wheat’s grain protein content, grain starch content, and grain hardness
Source: Sci Rep. 2020 Jul 27;10:12541. doi: 10.1038/s41598-020-69381-5 (PMC7385145; doi:10.1038/s41598-020-69381-5)
Supplement: Supplementary file 1 — Supplementary file1 (PDF 1567 kb) [file 41598_2020_69381_MOESM1_ESM.pdf]

Article type: **Research article**

Prospects of GWAS and predictive breeding for European winter wheat's grain protein content, grain starch content, and grain hardness

Quddoos H. Muqaddasi[1,†,*], Jonathan Brassac[1], Erhard Ebmeyer[2], Sonja Kollers[2], Viktor Korzun[2], Odile Argillier[3], Gunther Stiewe[4,‡], Jörg Plieske[5], Martin W. Ganal[5], and Marion S. Röder[1]

[1] Leibniz Institute of Plant Genetics and Crop Plant Research (IPK), Corrensstraße 3, D-06466 Stadt Seeland OT Gatersleben, Germany

[2] KWS LOCHOW GmbH, D-29303 Bergen, Germany

[3] Syngenta France S.A.S., F-78910 Orgerus, France

[4] Syngenta Seeds GmbH, D-32107 Bad Salzuflen, Germany

[5] TraitGenetics GmbH, Am Schwabeplan 1b, D-06466 Stadt Seeland OT Gatersleben, Germany

† Present address: European Wheat Breeding Center, BASF Agricultural Solutions GmbH, Am Schwabeplan 8, D-06466 Stadt Seeland OT Gatersleben, Germany

‡ Present address: SaKa Beteiligungsgesellschaft mbH, Albert-Einstein-Ring 5, D-22761 Hamburg, Germany

* Corresponding author: Quddoos H. Muqaddasi (muqaddasi@ipk-gatersleben.de)

Supplementary figures

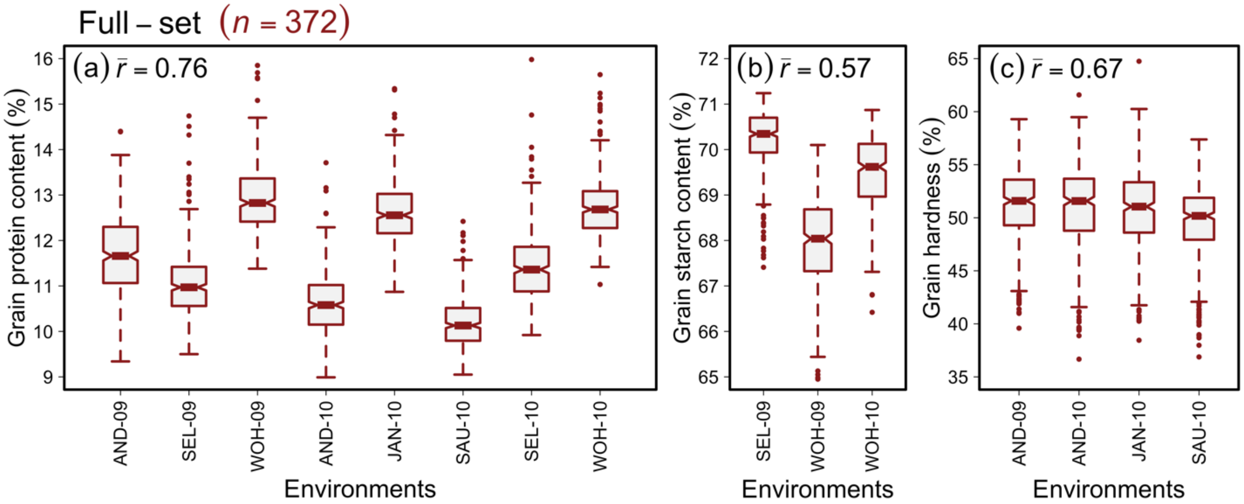

Figure S1. Environment specific phenotypic distribution of the investigated traits in a panel of 372 elite European winter wheat varieties. (**a**) Grain protein content, (**b**) grain starch content and (**c**) grain hardness. $\bar{r}$ denotes the average trait correlation calculated across environments based on eq. 3 in the manuscript.

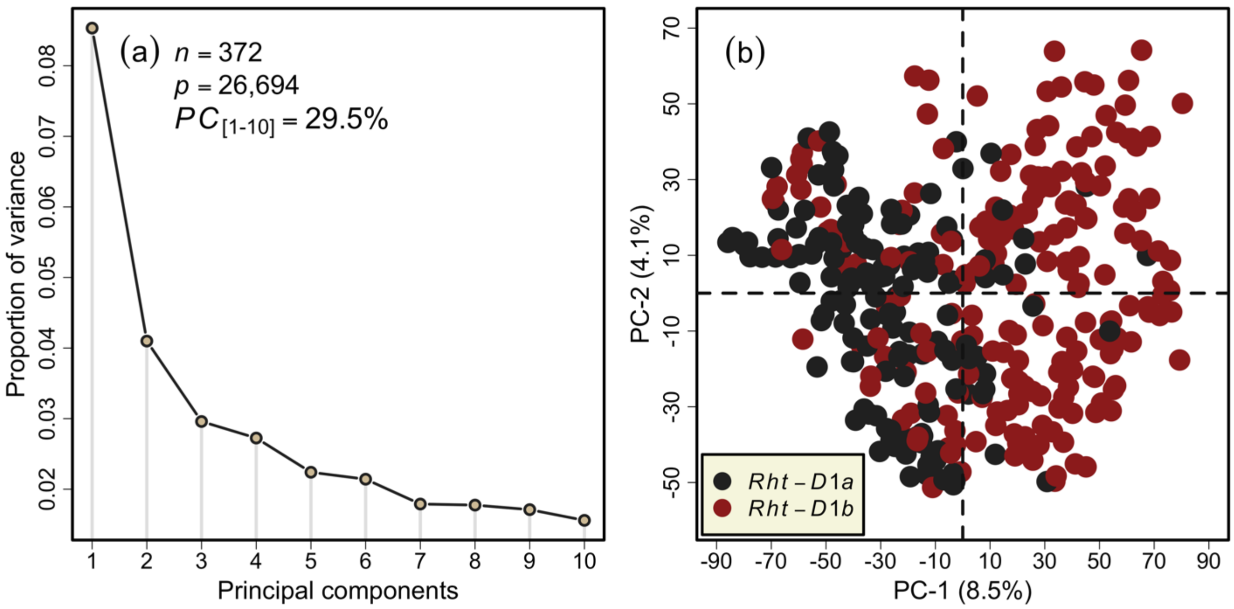

Figure S2. Principal component (PC) analysis on the wheat marker loci combined from the 35k and 90k single nucleotide polymorphism arrays. (**a**) Scree plot showing the first ten PCs and their corresponding proportion of variance, (**b**) scatterplot showing the absence of pronounced sub-clustering among the investigated wheat varieties. Different colors represent the *Rht-D1* alleles. n and p denote the number of varieties and the marker genotypes used in the analysis, respectively.

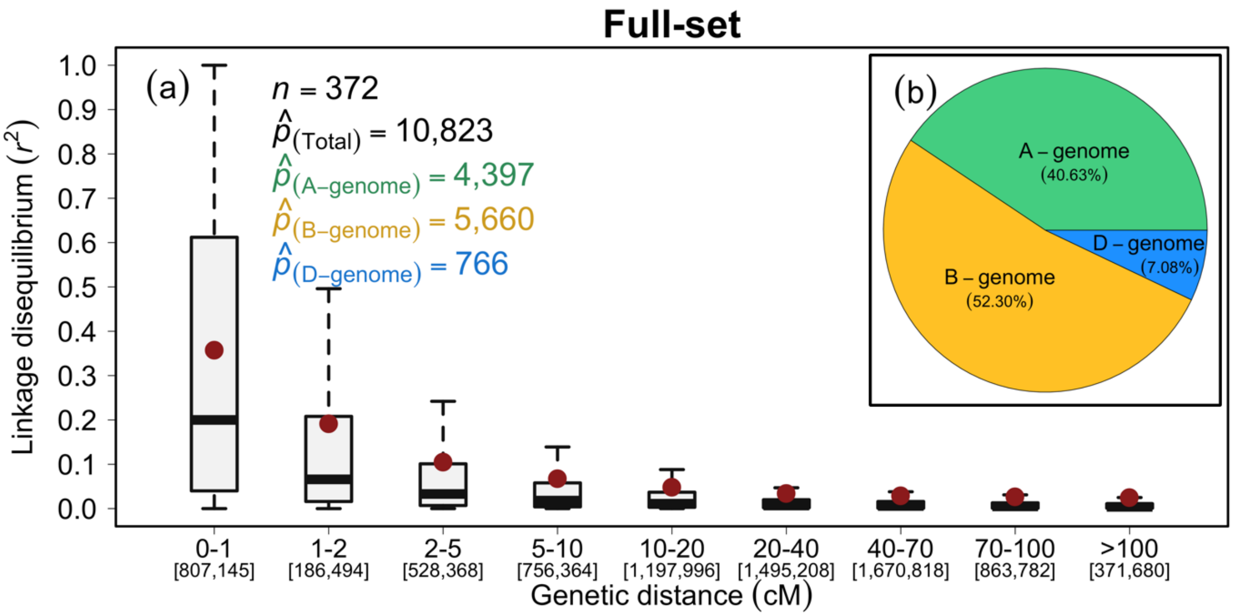

Figure S3. Genome-wide decay of linkage disequilibrium (LD; r^2) as a function of the genetic map distance (cM) between the maker loci in the population of European winter wheat varieties. (**a**) Boxplots represent the LD-decay, (**b**) sub-genome-wise distribution of the mapped marker loci. Red dots within the boxplots represent the mean values of the corresponding box. Th numbers on the second row of x-axis represent the number of marker-pair present in the corresponding genetic distance. n and $\hat{p}$ denote the number of varieties and mapped marker loci, respectively.

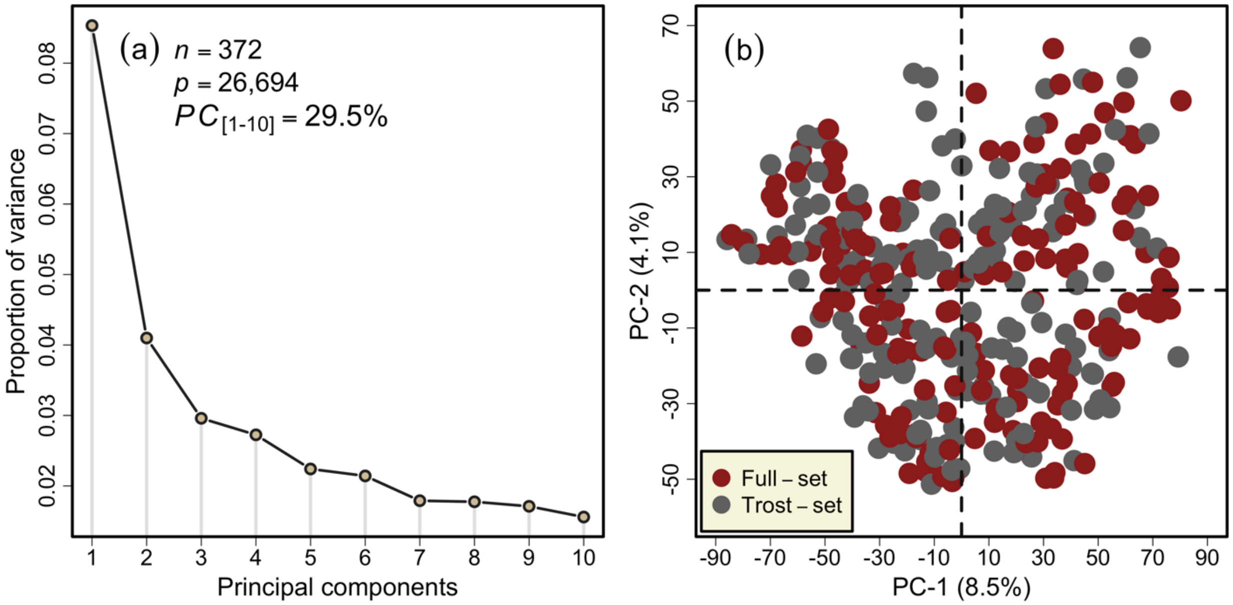

Figure S4. Principal component (PC) analysis of wheat varieties based on the high-quality marker loci combined from the 35k and 90k single nucleotide polymorphism arrays *plus* 27 candidate-genes markers. (**a**) Scree plot showing the first ten PCs and their corresponding proportion of variance, (**b**) scatterplot showing the absence of pronounced sub-clustering among the investigated wheat varieties. Different colors represent different wheat panels, *viz*., full-set and trost-set. n and p denote the number of varieties and the marker genotypes used in the analysis, respectively.

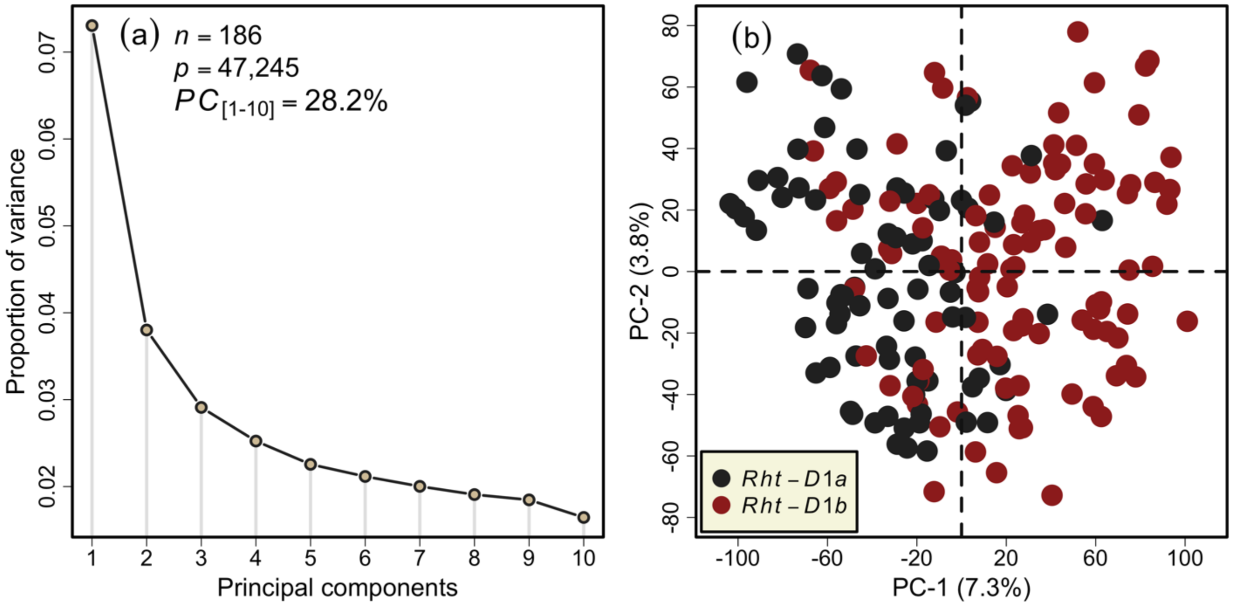

Figure S5. Principal component (PC) analysis of wheat TROST-set varieties based on high-quality marker loci combined from the 35k, 90k, 135k, single nucleotide polymorphism arrays *plus* 27 candidate-genes markers. (**a**) Scree plot showing the first ten PCs and their corresponding proportion of variance, (**b**) scatterplot showing the absence of pronounced sub-clustering among the investigated wheat varieties. Different colors represent the *Rht-D1* alleles. n and p denote the number of varieties and the marker genotypes used in the analysis, respectively.

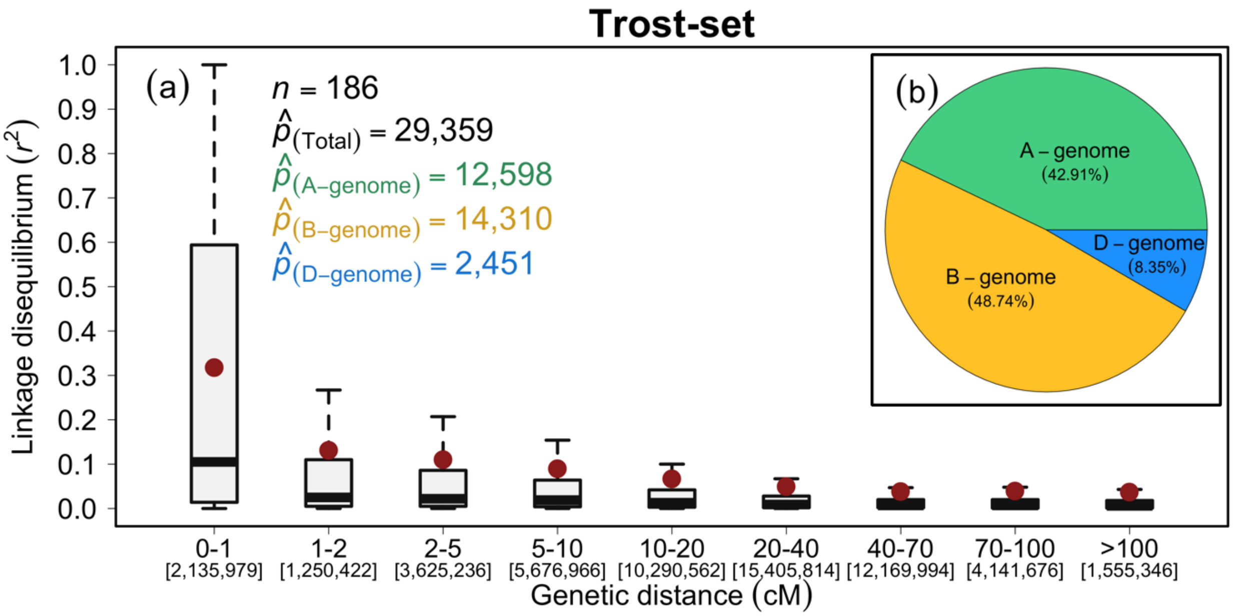

Figure S6. Genome-wide decay of linkage disequilibrium (LD; r^2) as a function of the genetic map distance (cM) between the maker loci in the representative (Trost-set) population of European winter wheat varieties. (**a**) Boxplots represent the LD-decay, (**b**) sub-genome-wise distribution of the mapped marker loci. Red dots within the boxplots represent the mean values of the corresponding box. Th numbers on the second row of x-axis represent the number of marker-pair present in the corresponding genetic distance. n and $\hat{p}$ denote the number of varieties and mapped marker loci, respectively.

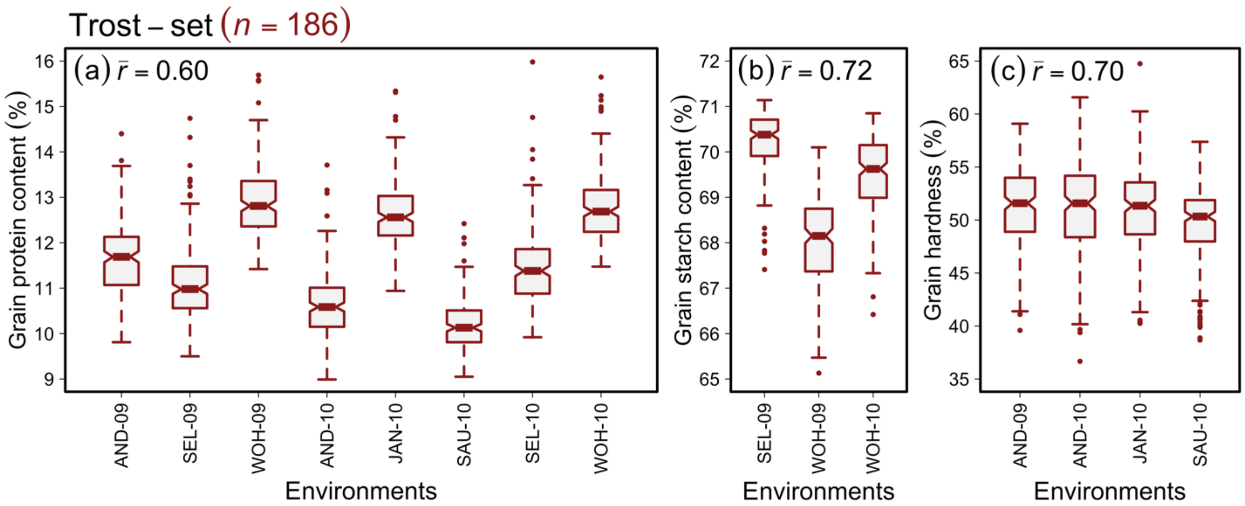

Figure S7. Environment specific phenotypic distribution of the investigated traits in the Trost-set comprising of 186 representative registered European wheat varieties. (**a**) Grain protein content, (**b**) grain starch content and (**c**) grain hardness. $\bar{r}$ denotes the average trait correlation calculated across environments based on eq. 3 in the manuscript.

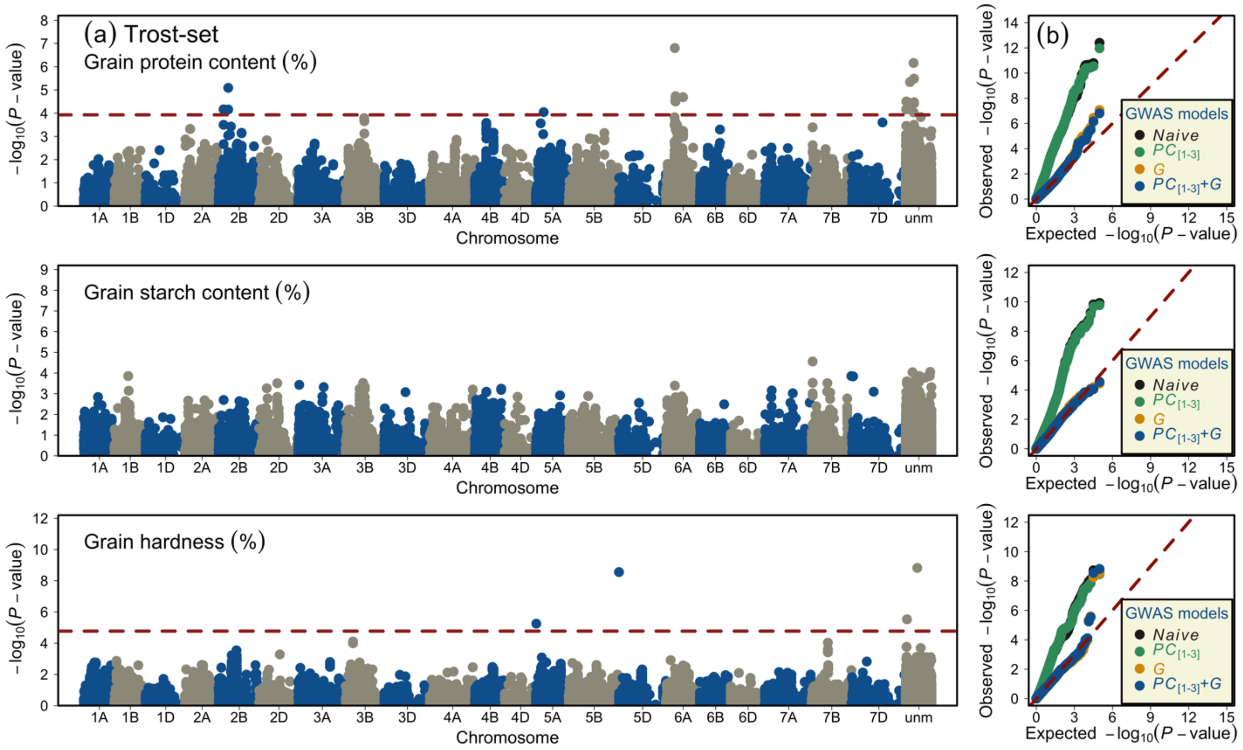

Figure S8. Summary of genome-wide association studies (GWAS) of investigated traits namely grain protein content, grain starch content and grain hardness in a panel of 186 registered wheat varieties. (**a**) Manhattan plots show the distribution of marker significance ($-\log_{10} P - \mathrm{value}$) along wheat chromosomes. The dashed red line indicates the significance threshold based on false discovery rate (FDR) of $P < 0.20$. (**b**) Quantile-quantile plots show the distribution of observed versus expected (red dashed line) $-\log_{10}(P - \mathrm{value})$. The naïve represents the GWAS without correction for population structure, the $PC_{[1\text{-}3]}$ represents the GWAS with population structure corrected with the first three principal components (*PC*), the *G* represents the GWAS with familial relatedness corrected with a genomic relationship matrix (*G*), and the $PC_{[1\text{-}3]}+G$ represents the GWAS corrected with both $PC_{[1\text{-}3]}$ and *G* matrix. The different GWAS models are color coded, and the results of $PC_{[1\text{-}3]}+G$ model are displayed in the Manhattan plots for individual traits.
